# Computed Tomography Structured Reporting in the Staging of Lymphoma: A Delphi Consensus Proposal

**DOI:** 10.3390/jcm10174007

**Published:** 2021-09-04

**Authors:** Vincenza Granata, Silvia Pradella, Diletta Cozzi, Roberta Fusco, Lorenzo Faggioni, Francesca Coppola, Roberta Grassi, Nicola Maggialetti, Duccio Buccicardi, Giorgia Viola Lacasella, Marco Montella, Eleonora Ciaghi, Francesco Bellifemine, Massimo De Filippo, Marco Rengo, Chandra Bortolotto, Roberto Prost, Carmelo Barresi, Salvatore Cappabianca, Luca Brunese, Emanuele Neri, Roberto Grassi, Vittorio Miele

**Affiliations:** 1 Division of Radiology, Istituto Nazionale Tumori IRCCS Fondazione Pascale—IRCCS di Napoli, 80131 Naples, Italy; v.granata@istitutotumori.na.it; 2 Italian Society of Medical and Interventional Radiology (SIRM), SIRM Foundation, 20122 Milan, Italy; pradellas@aou-careggi.toscana.it (S.P.); cozzid@aou-careggi.toscana.it (D.C.); roberta.grassi@policliniconapoli.it (R.G.); emanuele.neri@unipi.it (E.N.); roberto.grassi@unicampania.it (R.G.); vmiele@sirm.org (V.M.); 3 Division of Radiology, Azienda Ospedaliera Universitaria Careggi, 50134 Florence, Italy; 4 Medical Oncology Division, Igea SpA, 80100 Napoli, Italy; r.fusco@igeamedical.com; 5 Academic Radiology, Department of Translational Research, University of Pisa, 56126 Pisa, Italy; 6 Department of Radiology, IRCCS Azienda Ospedaliero-Universitaria di Bologna, 40138 Bologna, Italy; francesca_coppola@hotmail.com; 7 Division of Radiology, Università degli Studi della Campania Luigi Vanvitelli, 80138 Naples, Italy; giorgialacasella@gmail.com (G.V.L.); marco.montella@unicampania.it (M.M.); salvatore.cappabianca@unicampania.it (S.C.); 8 Section of Radiodiagnostics, DSMBNOS, “Aldo Moro” University, 70124 Bari, Italy; n.maggialetti@gmail.com; 9 Imaging Department, San Paolo Hospital, 17100 Savona, Italy; buccicardiduccio@hotmail.com; 10 Exprivia Spa, 70056 Bari, Italy; eleonora.ciaghi@exprivia.com (E.C.); francesco.bellifemine@exprivia.com (F.B.); 11 Unit of Radiologic Science, Department of Medicine and Surgery, University of Parma, Maggiore Hospital, 43126 Parma, Italy; massimo.defilippo@unipr.it; 12 Department of Radiological Sciences, Oncology and Pathology, Sapienza University of Rome—I.C.O.T. Hospital, 04100 Latina, Italy; marco.rengo@gmail.com; 13 Department of Radiology, I.R.C.C.S. Policlinico San Matteo Foundation, 27100 Pavia, Italy; chandra.bortolotto@gmail.com; 14 Radiology Unit, Azienda Ospedaliera Brotzu, 09134 Cagliari, Italy; rprost@sirm.org; 15 Diagnostic Imaging Section, Department of Medical and Surgical Sciences & Neurosciences, Siena University Hospital, 53100 Siena, Italy; carminev80@alice.it; 16 Department of Medicine and Health Sciences “V. Tiberio”, University of Molise, 86100 Campobasso, Italy; lucabrunese@libero.it

**Keywords:** radiology report, free text report, structured report, lymphoma, computed tomography

## Abstract

Structured reporting (SR) in radiology is becoming increasingly necessary and has been recognized recently by major scientific societies. This study aims to build structured CT-based reports for lymphoma patients during the staging phase to improve communication between radiologists, members of multidisciplinary teams, and patients. A panel of expert radiologists, members of the Italian Society of Medical and Interventional Radiology (SIRM), was established. A modified Delphi process was used to develop the SR and to assess a level of agreement for all report sections. The Cronbach’s alpha (Cα) correlation coefficient was used to assess internal consistency for each section and to measure quality analysis according to the average inter-item correlation. The final SR version was divided into four sections: (a) Patient Clinical Data, (b) Clinical Evaluation, (c) Imaging Protocol, and (d) Report, including *n* = 13 items in the “Patient Clinical Data” section, *n* = 8 items in the “Clinical Evaluation” section, *n* = 9 items in the “Imaging Protocol” section, and *n* = 32 items in the “Report” section. Overall, 62 items were included in the final version of the SR. A dedicated section of significant images was added as part of the report. In the first Delphi round, all sections received more than a good rating (≥3). The overall mean score of the experts and the sum of score for structured report were 4.4 (range 1–5) and 1524 (mean value of 101.6 and standard deviation of 11.8). The Cα correlation coefficient was 0.89 in the first round. In the second Delphi round, all sections received more than an excellent rating (≥4). The overall mean score of the experts and the sum of scores for structured report were 4.9 (range 3–5) and 1694 (mean value of 112.9 and standard deviation of 4.0). The Cα correlation coefficient was 0.87 in this round. The highest overall means value, highest sum of scores of the panelists, and smallest standard deviation values of the evaluations in this round reflect the increase of the internal consistency and agreement among experts in the second round compared to first round. The accurate statement of imaging data given to referring physicians is critical for patient care; the information contained affects both the decision-making process and the subsequent treatment. The radiology report is the most important source of clinical imaging information. It conveys critical information about the patient’s health and the radiologist’s interpretation of medical findings. It also communicates information to the referring physicians and records this information for future clinical and research use. The present SR was generated based on a multi-round consensus-building Delphi exercise and uses standardized terminology and structures, in order to adhere to diagnostic/therapeutic recommendations and facilitate enrolment in clinical trials, to reduce any ambiguity that may arise from non-conventional language, and to enable better communication between radiologists and clinicians.

## 1. Introduction

Lymphomas are very common malignant tumors, affecting children, young and older adults, and account for 5–6% of all malignancies. Hodgkin lymphoma (HL) and non-Hodgkin lymphoma (NHL) are the third most common malignant tumors in children [1]. In the 2017 World Health Organization (WHO) classification, more than 80 mature lymphoma entities are recognized, grouped into three major categories: B-cell neoplasms, T-cell and NK-cell neoplasms, and Hodgkin lymphomas (HLs). The disease entities are listed according to predominant clinical presentation (predominantly disseminated diseases that often involve bone marrow and may be leukemic, primary extra-nodal lymphomas, and predominantly nodal diseases, which are often disseminated and be interpreted in the light of clinical and histopathological features) [2,3,4]. The availability of more effective therapies for lymphoma and the increasingly sensitive and specific technologies for disease assessment provide a rationale for updated patient evaluation, staging, and response criteria. These should be unambiguous, universally applicable, and facilitate the comparison of patients and results among studies and the evaluation of new therapies [2,3,4]. The diagnosis of lymphoma depends on morphology, immunohistochemistry, and flow cytometry reviewed by an experienced lymphoma pathologist and, where appropriate, molecular studies to accurately categorize the lymphoma. A fine-needle aspirate is inadequate for initial diagnosis. An incisional or excisional biopsy is preferred to provide adequate tissue for these examinations. Staging defines disease location and extent, suggests prognostic information, allows comparisons among studies, and provides a baseline against which response or disease progression can be compared. Initial staging criteria were designed primarily for HL and were superseded by the Ann Arbor classification [2,3,4]. The Ann Arbor classification subdivides patients according to the absence or presence of disease-related symptoms. Following extensive experience with this classification, and recognizing the progress made (especially in imaging techniques), a workshop was held at the 11th International Conference on Malignant Lymphoma (ICML) in Lugano, Switzerland, in June 2011. The aims were to develop universally accepted, unambiguous, improved staging and response criteria for HL and NHL, relevant for community physicians, investigator-led trials, cooperative group and registration trials that would permit improved lymphoma patient evaluation, enhance comparisons amongst studies, and simplify the evaluation of new therapies [2,3,4].

Imaging in hematological tumors has evolved extensively over the past several decades. 18F-fluorodeoxyglucose positron emission tomography/computed tomography (18F-FDG-PET/CT) is currently essential for accurate staging and for early and late therapy response assessment. The widely adopted visual Deauville 5-point scale and Lugano Classification recommendations have recently standardized PET scans interpretation and allowed improvement in the management of lymphoma patients [5]. The CT as part of a PET-CT scan may be performed with contrast enhancement (ceCT) at full dose to obtain high quality images, or without contrast using a lower dose. ceCT may identify additional findings and improve detection of abdominal or pelvic disease [6]. In addition, small errors in the measurement of FDG uptake in tumor may occur with contrast media, because of an effect on attenuation correction, causing FDG uptake to be overestimated in the mediastinum and liver by 10% to 15% [7]. Several scientific societies (e.g., the European Association of Nuclear Medicine, the Society of Nuclear Medicine, and the Radiological Society of North America) recommend that patients undergo separate ceCT before PET-CT [6]. PET-CT is preferred for staging of FDG-avid lymphomas, whereas CT is preferred for the other lymphomas. Moreover, CT identifies more hilar nodes and may better discriminate between a single large nodal mass and an aggregate of individual nodes. Contrast-enhanced CT should be included for a more accurate measurement of nodal size and in the setting of compression/thrombosis of central/mediastinal vessels. Contrast-enhanced CT is also preferred for radiation planning. Variably FDG-avid histologies should be staged with CT [5,6,7].

An accurate reporting of imaging data to referring physicians is critical for patient care, as the information contained affects both the decision-making process and the subsequent treatment. The radiology report is the most important source of clinical imaging information, because it conveys critical information about the patient’s health and the radiologist’s interpretation of medical findings, communicates information to the referring physicians, and records that information for future clinical and research use. Although the efforts to structure some radiology reports via information through predefined templates are beginning to bear fruit, a large portion of radiology report information is entered in free text format. Free text reporting (FTR) is a major obstacle to the rapid extraction and subsequent use of radiological information by clinicians, researchers, and healthcare information systems [8,9,10,11]. In addition, inconsistencies regarding content, style, and presentation can hamper information transfer and decrease the clarity of the reports, which can in turn adversely affect the extraction of the required key information by the referring physician. At worst, the resulting communication errors can lead to incorrect diagnosis, delayed initiation of adequate treatment, or adverse patient outcomes [8]. Recently, the use of structured reporting (SR) has been recommended by several medical societies [8,9,10,11,12,13,14,15,16]. SR could offer several advantages over conventional narrative reporting, including a higher standardization of reporting style and lexicon, greater consistency and reproducibility of reports, full integration with hospital IT systems (along with the possibility of performing large-scale data mining), shorter reporting time, potential reduction of errors, and improved communication with other radiologists and referring clinicians [8,9,10,11,12,13,14,15,16]. To this regard, oncologists have been shown to prefer SR over conventional narrative reporting owing to its ability to convey information in a clearer and more standardized manner, and Shoeppe et al. reported that SR of CT examinations for primary staging in patients with diffuse large B-cell lymphoma adds clinical value compared to narrative reporting by increasing completeness of reports, facilitating information extraction, and improving patient management [16]. The main objectives for a shift from FTR to SR focus on three key features: quality, datafication/quantification, and accessibility [8]. In a position paper on radiological SR, the European Society of Radiology (ESR) has made a valuable contribution to the understanding of SR and its implementation, clearly describing the need for SR in clinical practice by addressing (a) its requirements and (b) implementation strategies [8]. It also stated that “the need to use uniform language and structure to accurately discuss findings in radiology is the basis for developing the concept of structured reporting” [8,16]. The use of templates in SR provides a checklist as to whether all relevant items are addressed. Moreover, thanks to this “structure”, SR allows the association of radiological data and other key clinical features, paving the way to personalized medicine. As regards accessibility, it is well known that radiology reports are a rich source of data for research. Therefore, thanks to this feature, it is possible to extract automated data.

Despite its acknowledged advantages, SR has not yet become established in the radiological routine. However, given the aforementioned advantages that SR would be likely to bring to radiology and other medical specialties, major scientific societies have striven to encourage its widespread adoption in radiological practice, including the use of standard templates by the Radiological Society of North America (RSNA) and the joint RSNA/ESR Reporting Initiative aimed at translating RSNA templates into a variety of European languages. In this scenario, the Italian Society of Medical and Interventional Radiology (SIRM) has created an Italian warehouse of SR templates that can be freely accessible by all SIRM members, with the purpose of using them routinely in a clinical setting [12].

As cancer therapy has rapidly evolved in the last decade with the availability of evermore sophisticated and individualized treatment options, accurate staging is of major importance to avoid over- and under-treatment. The implementation of a structured decision-tree template to guide the radiologist in lymphoma staging may potentially improve the quality of radiological reports with regard to completeness of information, comprehensibility and guidance for patient management. In this paper, we present a SR model based on CT examinations that defines and summarizes the clinical and radiological data of patients with lymphoma.

## 2. Materials and Methods

### 2.1. Expert Panel

A multi-round consensus-building Delphi exercise was performed to develop a comprehensive focused SR template for CT staging of patients with lymphoma, as a result of a critical discussion between expert radiologists.

A SIRM radiologist with experience in lymphoproliferative tumors created the first draft of the SR. A working team of 15 experts was then set up, including members from the SIRM Italian College of Diagnostic Imaging in Oncology Radiologists and the SIRM Foundation. Their aim was to revise the initial draft iteratively, trying to reach a final consensus on SR.

### 2.2. Selection of the Delphi Domains and Items

All the experts reviewed literature data on the main scientific databases (including PubMed, Scopus, and Google Scholar) to assess papers on CT staging in lymphoma patients and radiological SR published from December 2000 to May 2021. The full text of the selected studies was reviewed by all panelists, and each of them developed and shared the list of Delphi items via emails and/or teleconferences.

Two Delphi rounds were performed. During the first round, each panelist contributed independently to refining the SR draft by means of online meetings or email exchanges. The level of panelist agreement for each SR model was tested in the second Delphi through a Google Form questionnaire shared by email. Each expert expressed his/her individual comments for each specific template section by using a five-point Likert scale (1 = strongly disagree, 2 = slightly disagree, 3 = slightly agree; 4 = generally agree, 5 = strongly agree).

After the second Delphi round, the final version of the SR was generated on the dedicated RSNA website (radreport.org) by using a T-Rex template format, in line with IHE (Integrating Healthcare Enterprise) and the MRRT (Management of Radiology Report Templates) profiles, accessible as open-source software, with the technical support of Exprivia™ (Exprivia SpA, Bari, Italy). This determines both the format of radiology report templates [using version 5 of Hypertext Markup Language (HTML5)] and the transporting mechanism to request, retrieve, and stock these schedules. The radiology report was structured by using a series of “codified queries” integrated in the T-Rex editor’s preselected sections [17].

### 2.3. Statistical Analysis

Each section of SR was analyzed by calculating mean and standard deviation values. Moreover, the sum of scores for each section was calculated. A mean value ≥3 was considered good, whereas a mean value ≥4 was considered excellent.

The consistency of the panelist evaluations for each section of the SR was calculated with the Cronbach’s alpha (Cα) correlation coefficient [18,19]. A Cα coefficient ≥0.9 was considered excellent, a Cα ≥0.8 good, a Cα ≥0.7 acceptable, a Cα ≥0.6 questionable, a Cα ≥0.5 poor, and a Cα <0.5 unacceptable.

Data analysis was performed using Statistic Toolbox of Matlab (The MathWorks, Inc., Natick, MA, USA).

## 3. Results

### 3.1. Structured Report

The final version of the SR (Supplementary Material) was divided into four sections: (a) Patient Clinical Data, (b) Clinical Evaluation, (c) Imaging Protocol, and (d) Report, including *n* = 13 items in the “Patient Clinical Data” section, *n* = 8 items in the “Clinical Evaluation” section, *n* = 9 items in the “Imaging Protocol” section, and *n* = 32 items in the “Report” section, respectively. Overall, 62 items were included in the final version of the SR. A dedicated section for key images was added as part of the report.

The “Patient Clinical Data” section included patient clinical data, personal or family history of cancer, lifestyle, and dietary habits. In this section, we included the item “Allergies” to drugs and contrast agents.

The “Clinical Evaluation” section collected data on prior imaging examination or biopsy results, clinical presentation, CEA level, blood count, serum creatinine, liver function, and virology tests.

The “Imaging Protocol” section included data on the CT equipment used, the number of detector rows and whether it was single and/or dual energy, reconstruction algorithm(s) and slice thickness. In addition, we collected data on contrast medium protocol, including data on the post-contrast acquisition(s), active principle of contrast agent, commercial name, dosage, flow rate, concentration, and ongoing adverse events. In addition, we included data on bowel preparation and the class of radiation exposure.

The “Report” section included data on the following:

1. Lesion site (e.g., lymph node disease, bulky disease, spleen or extra-nodal disease). For nodal disease we clarified the site, according to the stage: limited disease (stage I-II) or advanced disease (stage III-IV).
2. Size, i.e., largest dimension on axial plane (mm) and dimension of the axis perpendicular to the largest diameter (mm).
3. CT appearance (areas of contrast enhancement and areas of necrosis/colliquation).
4. Relationship with neighboring structures.

In this section we also included data on non-measurable lesions, on selected target lesions, total lesion burden, complications, incidental findings unrelated to tumors, and conclusions.

### 3.2. Consensus Agreement

Table 1 shows single scores and sum of scores of the 15 panelists for SR in the first Delphi round, whereas Table 2 reports the same scores related to the second Delphi round.

In the first round, all sections received more than a good rating (≥3). The overall mean score of the experts and the sum of score for structured report were 4.4 (range 1–5) and 1524 (mean value of 101.6 and standard deviation of 11.8), respectively. The Cα correlation coefficient was 0.89 in the first round and hence was considered good.

In the second round, all sections received more than an excellent rating (≥4). The overall mean score of the experts and the sum of scores for structured report were 4.9 (range 3–5) and 1694 (mean value of 112.9 and standard deviation of 4.0), respectively. The Cα correlation coefficient was 0.87 in this round and hence was considered good. The highest overall means value, the highest sum of scores of the panelists, and the smallest standard deviation values of the evaluations in this round reflect the increase of the internal consistence and agreement among experts in the second round compared to the first round.

## 4. Discussion

To the best of our knowledge, this was the first time that a group of experts promoted the creation of a CT-based SR for the staging of lymphoproliferative tumors, based on a multi-round consensus-building Delphi exercise.

The final SR version was divided into four sections: (a) Patient Clinical Data, (b) Clinical Evaluation, (c) Imaging Protocol and (d) Report, including *n* = 62 items in the final version of the SR. Even if this model may seem too long and complicated (possibly slowing down the workflow of a radiologist), it is necessary to highlight that the only section required of it is the “report section”, whereas all other sections are optional. Additionally, considering that not all records may be accessible, these are open fields that could also be filled in at a later time. Patient data can also be automatically imported from the patient’s electronic file.

In the first Delphi round, all sections received more than a good rating (≥3). The Cα correlation coefficient was 0.89 in the first round and hence was considered good.

The weakest sections were “Patient Clinical Data” and “Clinical Evaluation”. These results were connected to the idea that these sections are too long, slowing down the daily workflow. However, after call conferences and mail exchanges, and once the optionality of the sections had been clarified, all panelists expressed their agreement.

In the second Delphi round, all sections received more than an excellent rating (≥4). The Cα correlation coefficient was 0.87 in this round and hence was considered good. The highest overall mean value, the highest sum of scores of the panelists, and the smallest standard deviation values of the evaluations in this round reflect the increase of the internal consistency and agreement among experts in the second round compared to the first round.

The “Patient Clinical Data” section included patient clinical data, personal or family history of cancer, lifestyle, and dietary habits. In this section, we included the item “Allergies” to drugs and contrast agents. The “Clinical Evaluation” section collected data on prior imaging examination or biopsy results, clinical presentation, CEA level, blood count, serum creatinine, liver function, and virology tests. Thanks to this framework, this template permits the connection of radiological findings with clinical data, thus allowing for automated data extraction, which may help validate the relevance of imaging biomarkers to enable personalized medicine. In recent years, through its ability to assemble and quickly analyze enormous volumes of data generated by imaging studies, artificial intelligence (AI) has begun to transform the practice of radiology. Throughout the field, applications leveraging AI are used to improve diagnostic accuracy, imaging consistency, workflow efficiency, and patient care by automating many formerly tedious, time consuming, and manually performed tasks [20,21,22,23,24]. Radiomics is an emerging field of radiology and can be coupled with AI. Radiomic features provide data on tumor phenotype as well as cancer microenvironment. Radiomics-derived parameters, when associated with other clinical pertinent data and correlated with outcomes data, can produce accurate robust evidence-based clinical-decision support systems (CDSS) [25,26,27,28,29,30,31,32]. The possibility to connect radiological and clinical data in the present SR template could create the basis for a large database, allowing not only epidemiological statistical analyses, but also the building of radiomics models [29]. In this context, the added value of genomic data could be used to develop radio-genomics models, which would be helpful considering the highest level of personalized risk stratification and the advanced process of precision medicine [33,34,35].

The “Imaging Protocol” section included data on the CT equipment used (e.g., single and/or dual energy), slice thickness, reconstruction algorithm(s), type of contrast medium (e.g., active principle, commercial name, dosage, flow rate, concentration), contrast study protocol, and ongoing adverse events. In addition, we included data on bowel preparation and the class of radiation exposure. The sharing of technical data, as well as the sharing of the study protocol used, allows an optimization of the study technique, both in terms of exposure dose and contrast dose [36,37,38]. In addition, using the same protocols allows not only comparison of data obtained in different clinical studies, but also a better diagnostic accuracy [39,40].

The “Report” section included data on lesion site (e.g., Lymph node disease, Bulky disease, Spleen or Extra-nodal disease). For nodal disease we clarified the site, according to: (a) stage [limited disease (stage I-II) or advanced disease (stage III-IV)], (b) lesion size [largest dimension on axial plane (mm) and dimension of the axis perpendicular to the largest diameter (mm)], (c) CT appearance of the lesions (areas of contrast enhancement and areas of necrosis/colliquation), and (d) relationship with neighboring structures. In addition, in this section we included data on non-measurable lesions, on selected target lesions, total lesion burden, complications, incidental findings unrelated to tumors, and conclusions. The opportunity to use a template guiding the radiologists during their practice allows the description of all radiological findings (indispensable for a correct staging of the disease), which could be omitted by pure distraction using FTR. Schoeppe et al. showed that the advantages of SRs included faster extraction of relevant findings, better comparability, improvement of diagnostics and treatment planning, fewer radiologist consultations required, and improved clarity [16]. Misinterpretation of findings potentially leading to up- or downstaging and inadequate therapy, less communication between radiologist and practitioner, and the higher professionalism of the radiologist needed regarding evaluation of findings and staging were seen as disadvantages of SRs by two reviewers [16]. As an advantage, one reviewer pointed out that using an anatomical layout, relationships (infiltration *per continuitatem*) are sometimes easier to understand with FTR and important findings can be emphasized. Disadvantages of FTR noted by two reviewers comprised high interobserver variability, incomparability of findings, no quality characteristic, notably more confusing layout, and loss of important information [16]. According to Schoeppe et al., SR contained significantly more, often explicit, data on organs affected than FTR. Findings that allowed for Ann-Arbor classification of disease stage were significantly more often present in SR compared to FTR. The SR also included significantly more often reference lesions necessary for monitoring disease progression. Differences between SR and FTR regarding missing key features were significant for extra-nodal involvement and bulky disease. Reporting of the location of affected lymph node regions was incomplete in 2% of SRs and 5% of FTRs. The number of affected lymph node regions was missing in 27% of SRs and 30% of FTRs. Measurements of lymph nodes as reference lesions were less frequently absent in SRs than FTRs (28% vs. 42%). Six percent of SRs did not comment on involvement of the spleen compared to 14% of FTRs. Reporting on extra-nodal involvement was significantly more often missing in FTRs, 38% vs. 9% in SRs (*p* < 0.001). In addition, several authors have analyzed the impact of the SR in minimizing errors in radiology, demonstrating that a checklist allows not only the identification of more data, but also the adoption of the most appropriate therapeutic approach [41,42,43,44,45]. A retrospective analysis of 3000 MRI studies showed that, in 28.5% of patients, the use of SR allowed the identification of extraspinal findings that were not included in the original FTR [41]. In fact, SR has been shown to improve the rate of diagnosis of incidental findings [42], as well as to improve the clinical impact on surgical planning for pancreatic and rectal carcinoma [43,44,45].

The present SR is based on standardized terminology and structures, features required in order to adhere to diagnostic–therapeutic recommendations and enrolment in clinical trials, to reduce any ambiguity that may arise from non-conventional language, and to enable better communication between radiologists and clinicians, configuring a third-level SR according to Weiss et al. [46].

Despite the promising results obtained, this study has several limitations. Firstly, the panelists were made up only of radiologists, so a multidisciplinary approach is lacking, while a multidisciplinary validation of SR would be more appropriate. Secondly, the panelists were from the same country, yet the contribution of experts from multiple countries should allow a broader sharing and an increase the template consistency. Thirdly, in this paper we did not assess the impact of the SR on the management of patients with lymphoproliferative tumors.

## 5. Conclusions

An accurate reporting of imaging data to referring physicians is critical for patient care, as the information contained in the radiological report affects both the decision-making process and the subsequent treatment. The radiology report is the most important source of clinical imaging information. It conveys critical information about patient health and the radiologist’s interpretation of medical findings. It also communicates information to the referring physicians and records that information for future clinical and research use. The present SR was generated based on a multi-round consensus-building Delphi exercise and uses standardized terminology and structures, in order to adhere to diagnostic/therapeutic recommendations and facilitate enrolment in clinical trials, to reduce any ambiguity that may arise from non-conventional language, and to enable better communication between radiologists and clinicians.

## Figures and Tables

**Table 1:** Single scores and sum of scores of 15 panelists for structured report in the first Delphi round.

Panelist #	A1. Anthropometric Data	A2. Personal Assessments	A3. Allergies and Adverse Reactions	B1. Clinical Presentation	B2. BOM	B3. Laboratory Tests	B4. Histology	C1. Exam Data	C2. Precontrast Scans	C3. Post-Contrast Scans	4. Dosimetric Data	C5. Use of Contrast Medium	C6. Adverse Events in Progress	D1. Lymph Node Locations	D2. Bulky Disease	D3. Spleen	D4. Extranodal Locations	D5. Non Measurable Injuries	D6. Bone Lesions	D7. Selected Target Lesions	D8. SPD (Sum Product Diameter) Calculation	D9. Conclusions	E1. Meaningful Key Images	Sum
1	3	3	4	4	4	4	4	4	4	5	4	5	4	5	5	5	5	5	5	5	5	5	5	102
2	5	5	4	3	3	3	3	5	3	3	1	5	5	5	5	4	5	5	5	4	5	5	5	96
3	3	3	5	5	5	5	5	5	5	5	5	5	5	5	5	5	5	5	5	5	5	5	5	111
4	4	4	4	4	4	4	3	4	4	4	4	4	4	4	4	4	3	4	4	4	4	4	5	91
5	5	5	5	5	5	5	5	5	4	5	4	5	5	5	5	5	5	5	5	5	5	5	5	113
6	5	5	5	5	5	5	5	5	5	5	5	4	5	5	5	5	5	5	5	5	5	5	5	114
7	4	3	5	5	5	2	5	4	1	1	5	5	5	5	5	4	5	3	5	5	5	5	5	97
8	3	3	3	2	2	2	2	4	3	2	5	5	5	3	4	5	3	3	3	3	2	3	5	75
9	5	5	5	5	5	5	5	5	3	3	5	5	5	5	5	5	5	5	5	5	5	5	5	111
10	4	5	5	4	5	5	4	5	5	5	5	5	5	5	5	5	5	5	5	5	5	5	5	112
11	5	5	5	5	5	5	5	5	5	5	5	5	5	5	5	5	5	5	5	5	5	5	5	115
12	4	3	4	5	2	3	5	4	5	5	5	2	5	3	4	5	3	3	3	3	2	3	5	86
13	3	5	5	5	5	5	5	5	4	3	5	5	5	5	2	3	4	5	4	3	3	2	5	96
14	2	3	5	4	5	5	5	5	4	2	5	5	5	3	5	5	5	3	5	4	5	5	2	97
15	5	4	5	5	5	4	5	5	5	5	5	4	5	3	5	5	5	5	5	3	5	5	5	108
Mean	4.0	4.1	4.6	4.4	4.3	4.1	4.4	4.7	4.0	3.9	4.5	4.6	4.9	4.4	4.6	4.7	4.5	4.4	4.6	4.3	4.4	4.5	4.8	101.6
Standard deviation	0.9	1.0	0.7	1.0	1.1	1.2	1.1	0.5	1.3	1.5	1.3	0.4	0.4	0.7	0.4	0.5	0.8	0.8	0.7	0.7	1.0	0.7	0.0	11.8

**Table 2:** Single scores and sum of scores of 15 panelists for structured report in the second Delphi round.

Panelist #	A1. Anthropometric Data	A2. Personal Assessments	A3. Allergies and Adverse Reactions	B1. Clinical Presentation	B2. BOM	B3. Laboratory Tests	B4. Histology	C1. Exam Data	C2. Precontrast Scans	C3. Post-contrast Scans	4. Dosimetric Data	C5. Use of Contrast Medium	C6. Adverse Events in Progress	D1. Lymph Node Locations	D2. Bulky Disease	D3. Spleen	D4. Extranodal Locations	D5. Non Measurable Injuries	D6. Bone Lesions	D7. Selected Target Lesions	D8. SPD (Sum Product Diameter) Calculation	D9. Conclusions	E1. Meaningful Key Images	Sum
1	5	5	5	5	5	5	5	5	5	5	5	5	5	5	5	5	5	5	5	5	5	5	5	115
2	5	5	5	5	5	5	5	5	5	5	5	5	5	5	5	5	5	5	5	5	5	5	5	115
3	5	3	5	3	3	3	4	5	4	4	5	5	5	5	5	5	5	5	5	4	5	5	5	103
4	5	5	5	5	5	5	5	5	5	5	5	5	5	5	5	5	5	5	5	5	5	5	5	115
5	3	4	5	5	4	5	5	4	5	5	4	5	5	5	5	4	5	3	4	5	5	5	5	105
6	5	5	5	5	5	5	5	5	5	5	5	5	5	5	5	5	5	5	5	5	5	5	5	115
7	5	5	5	5	5	4	5	5	4	4	5	5	5	4	5	5	5	5	5	4	4	5	5	109
8	5	5	5	5	5	5	5	5	5	5	5	5	5	5	5	5	5	5	5	5	5	5	5	115
9	5	5	5	4	5	5	5	5	5	5	5	5	5	5	5	5	5	4	5	5	5	5	5	113
10	5	5	5	5	5	5	5	5	5	5	5	5	5	5	5	5	5	5	5	5	5	5	5	115
11	5	5	5	5	5	5	5	5	5	5	5	5	5	5	5	5	5	5	5	5	5	5	5	115
12	4	5	5	5	5	5	5	5	5	5	5	5	5	5	5	5	5	5	5	5	5	5	5	114
13	5	5	5	5	5	5	5	5	5	5	5	5	5	5	5	5	5	5	5	5	5	5	5	115
14	5	5	5	5	5	5	5	5	5	5	5	5	5	5	5	5	5	5	5	5	5	5	5	115
15	5	5	5	5	5	5	5	5	5	5	5	5	5	5	5	5	5	5	5	5	5	5	5	115
Mean	4.8	4.8	5.0	4.8	4.8	4.8	4.9	4.9	4.9	4.9	4.9	5.0	5.0	4.9	5.0	4.9	5.0	4.8	4.9	4.9	4.9	5.0	5.0	112.9
Standard deviation	0.6	0.7	0.0	0.7	0.7	0.7	0.3	0.3	0.4	0.4	0.3	0.0	0.0	0.3	0.0	0.3	0.0	0.7	0.3	0.4	0.3	0.0	0.0	4.0

## Data Availability

The single scores and sums of scores of the 15 panelists for the first and second Delphi rounds were reported in full in Table 1 and Table 2.

## Supplementary Materials

The following are available online at https://www.mdpi.com/article/10.3390/jcm10174007/s1, The final SR template is available as Supplementary Materials in Word format.
