# Supplementary material for: Computed Tomography Structured Reporting in the Staging of Lymphoma: A Delphi Consensus Proposal
Source: J Clin Med. 2021 Sep 4;10(17):4007. doi: 10.3390/jcm10174007 (PMC8432477; doi:10.3390/jcm10174007)
Supplement: Supplementary file 1 [file jcm-10-04007-s001.zip › jcm-1349285-supplementary.pdf]

Supplementary Materials

PATIENT CLINICAL DATA

<table>
<tr><th>FIELD</th><th>DETAIL</th><th colspan="2">ADMITTED VALUES</th></tr>
<tr><td colspan="4">ANTHROPOMETRIC DATA</td></tr>
<tr><td>Weight</td><td></td><td colspan="2">(kg) [Numeric]</td></tr>
<tr><td>Height</td><td></td><td colspan="2">(cm) [Numeric]</td></tr>
<tr><td>BMI</td><td></td><td colspan="2">[Numeric] (automatically calculated)</td></tr>
<tr><td>BSA</td><td></td><td colspan="2">[Numeric] (automatically calculated)</td></tr>
<tr><td>Age</td><td></td><td colspan="2">(years) [Numeric]</td></tr>
<tr><td>Age range</td><td></td><td colspan="2">• < 50 years
• > 50 years</td></tr>
<tr><td colspan="4">PATIENT HISTORY</td></tr>
<tr><td rowspan="3">Family history of cancer
(visible only if “Yes” and repeatable)</td><td>Yes/No</td><td colspan="2"></td></tr>
<tr><td>Degree of kinship</td><td colspan="2">• Mother
• Father
• Brother(s)/sister(s)
• Maternal grandparent(s)
• Paternal grandparent(s)
• Uncle(s)/aunt(s)
• Other [free text]</td></tr>
<tr><td>Notes</td><td colspan="2">[free text]</td></tr>
<tr><td rowspan="2">Personal history of other cancers</td><td>Yes/No</td><td colspan="2"></td></tr>
<tr><td>Notes</td><td colspan="2">[free text]</td></tr>
<tr><td rowspan="5"><u>Lifestyle/dietary habits</u>
(visible only if “Yes” and repeatable)</td><td>Smoke</td><td colspan="2">Yes/No</td></tr>
<tr><td colspan="3"><u>SMOKING DETAILS</u> (visible only if Smoke = “Yes”)</td></tr>
<tr><td rowspan="3"></td><td>Smoker</td><td>• Current smoker
• Former smoker</td></tr>
<tr><td>Cigarette smoke</td><td>Yes/No</td></tr>
<tr><td>Number of daily cigarettes (if “Current smoker”)</td><td>• Light (< 15)
• Heavy (≥15)</td></tr>
</table>

		Smoking years	*[Numeric]*
		Years from cessation<br>(if “Former smoker”)	• ≤ 15<br>• >15
		Cigarettes per year (pack-year)<br>(if “Former smoker” or “Current smoker”)	*[Numeric]*<br>(automatically calculated)*<br><br>*(Number of daily cigarettes x smoking years / 20)
		Vaping	Yes/No
		Number of daily electronic cigarettes refills (if vaping = “Yes”)	*[Numeric]*
		Number of years<br>(if vaping = “Yes”)	*[Numeric]*
		Notes	*[free text]*
	High alcohol intake	Yes more than 1 glass/day for women and 2 glasses/day for men)<br><br>No	
	High meat intake	Yes (white or red meat intake more than 3 times/week)<br><br>No	
	High cured meat intake	Yes *(cured meat intake more than once a week)<br><br>No	
	Low vegetable intake	Yes *(less than 2 servings/day)<br><br>No	
	Low fruit intake	Yes (*less than 3 whole fruits/day)<br><br>No	
	Notes	*[free text]*	
**ALLERGIES AND ADVERSE REACTIONS**			
**Reported allergies**<br>**(visible only if “Yes” and repeatable)**	Yes/No		
	Type	• Drug-related (n of drugs)<br>• Contrast medium-related (n of contrast media)<br>• Drug-unrelated	
	Active principle/molecule<br><br>[if drug- or contrast medium-related allergy]	*[free text]*	

**PREVIOUS adverse reactions**<br>**(visible only if "Yes" and repeatable)**	Commercial name<br>[if drug- or contrast medium-related allergy]	*[free text]*
	Notes	*[free text]*
	Yes/No	
	Date	month/year [mm/yyyy]
	Type	Contrast medium-related / unrelated
	Degree	• Mild<br>• Moderate<br>• Severe
	Time of onset	• Early<br>• Late
	Notes	*[free text]*
**Antiallergic premedication**	Yes/No	
	Treatment	• Steroid<br>• Antihistamine
	Complete	Yes/No
	Notes	*[free text]*
**Nephroprotective protocol**	Yes/No	
	Complete	Yes/No
	Serum creatinine	*[Numeric]* (mg/dl)
	GFR (Glomerular Filtration Rate)	*[Numeric]* (ml/min)<br><br>https://www.merckmanuals.com/medical-calculators/GFR_CKD_EPI-it.htm (sex, race, age, serum creatinine)
	Notes	*[free text]*

CLINICAL EVALUATION

FIELD	DETAIL	ADMITTED VALUES
**CLINICAL INFORMATION**	Yes/No	
**Prior imaging tests**<br>**(visible only if "Yes" and repeatable)**	Type	• CT<br>• MRI<br>• Ultrasound<br>• PET-CT<br>• Other *[free text]*
	Date	*[dd/mm/yyyy]*
	Notes	*[free text]*
**CLINICAL PRESENTATION**		
**Patient general condition**	• Good<br>• Poor	
	Symptoms	• Painless lymphoadenopathies (e.g., neck, underarm, groin, etc.)<br>• Fever and/or night sweats<br>• Unexplainable weight loss for at least 6 months<br>• Itching and/or pain triggered by alcohol intake (HL)<br>• Other *[free text]*
**Biopsy**	Yes/No<br>If Yes:	
		• Bone marrow biopsy (date *[mm/yyyy]*)<br>• Other *[free text]*, date *[mm/yyyy]*)
	Diagnosis (incl. immunohistotype)	*[free text]*
**CEA level**		*[Numeric]*
**Blood count**		*[Numeric]*
**Serum creatinine**		*[Numeric]*
**Liver function**		• Normal<br>• Impaired
**Virology tests**		• HBV (+/-)

		● HCV (+/-)<br>● HIV (+/-)<br>● Other *[free text]*
	Notes	*[free text]*

IMAGING PROTOCOL

FIELD	DETAIL	ADMITTED VALUES
**IMAGING DATA**		
**Date of examination**		Date *[dd/mm/yyyy]*
**Clinical indication**	Primary staging	
**Scanner brand and model**		*[free text]*
**Scanning technique**	Number of detector rows	*[Numeric]*
	Precontrast scan<br>***(*details visible only if "Yes")***	Yes/No<br>Dual energy (Yes/No)<br>Slice thickness (mm) *[Numeric]*<br>Convolution kernel(s) *[free text]*<br>Body area *[multiple choice]*:<br>● abdomen<br>● chest<br>● neck<br>● brain
	Post-contrast scan<br>***(*details repeatable for each post-contrast scan)***	*[Numeric]*<br>Post-contrast phase(s) (arterial, venous, late)<br>Dual energy (Yes/No)<br>Slice thickness (mm) *[Numeric]*<br>Convolution kernel(s) *[free text]*<br>Body area *[multiple choice]*:<br>● abdomen<br>● chest

		• neck<br>• brain
**Bowel preparation**	Yes/No	If Yes, specify *[free text]*
**Radiation exposure**	Class of radiation exposure	*[Numeric]*
**CONTRAST MEDIUM**		
**Use of contrast medium**<br>**(visible only if "Yes")**	Si/No	
	Yes/No	
	Active principle	• Iobitridol<br>• Iodixanol<br>• Iohexol<br>• Iomeprol<br>• Iopromide<br>• Ioversol
	Commercial name	*[free text]*
	Volume	*[Numeric]* (ml)
	Flow rate	*[Numeric]* (ml/sec)
	Concentration	*[Numeric]* (mgI/ml)
**ADVERSE EVENTS**		
**ONGOING adverse events**<br>**(visible only if "Yes")**	Yes/No	
	Date and hour of event	*[dd/mm/yyyy, hour]*
	Degree	• Mild<br>• Moderate<br>• Severe
	Time of onset	• Early<br>• Late<br><br>Minutes *[Numeric]* (optional)
	Type	ALLERGIC / ALLERGIC-LIKE<br>**Mild**<br>• Sparse wheals/itch<br>• Skin edema<br>• Mild itching / feeling like "velvet in the throat"<br>• Nasal congestion

		• Sneezing<br>• Conjunctivitis<br>• Rhinorrhea<br><br>**Moderate**<br>• Diffuse wheals/intense itch<br>• Diffuse skin edema<br>• Facial edema without dyspnea<br>• Feeling of choking or hoarseness<br>• Wheezing / mild bronchospasm without hypoxia<br><br>**Severe**<br>• Dyspnea<br>• Erythema – diffuse mucocutaneous symptoms<br>• Laryngeal edema with stridor and/or hypoxia<br>• Wheezing / bronchospasm<br>• Significant hypoxia<br>• Anaphylactic shock (severe hypotension and brady-tachyarrhythmia)<br><br>NON-ALLERGIC<br>**Mild**<br>• Mild nausea/limited vomiting<br>• Transient chills / heat / redness<br>• Headache / dizziness / anxiety / altered taste<br>• Slight increase in blood pressure<br>• Self-limiting vasovagal reaction<br>**Moderate**<br>• Prolonged nausea/vomiting<br>• Elevated arterial blood pressure<br>• Isolated chest pain<br>• Vasovagal reaction<br><br>**Severe**<br>• Treatment-refractory vasovagal reaction<br>• Arrhythmia<br>• Convulsions<br>• Severe arterial hypertension<br><br>CONTRAST MEDIUM EXTRAVASATION

	Type of treatment	• Wait and see<br>• Drug therapy (specify in “Notes” field)<br>• Anesthesiologist’s intervention required
	Event resolution	• Spontaneous<br>• After treatment<br>• After hospitalization<br>• Other *[free text]*
	Notes	*[free text]*

REPORT

FIELD	DETAIL	ADMITTED VALUES
**DIAGNOSIS**<br>**PRIMARY TUMOR**<br>**(open following fields depending on the “Site” of primary tumor)**		
	Site	• Lymph node disease<br>• Bulky disease<br>• Spleen<br>• Extranodal disease
**Lymph node disease**	Number	*[Numeric]*
	Site<br>(up to 2 target lesions can be selected)	• Limited disease (stage I-II) / advanced disease (stage III-IV)<br>If “Limited disease”:<br>○ Supradiaphragmatic sites *[free text]*<br>○ Subdiaphragmatic sites *[free text]*<br>If “Advanced disease”:<br>○ site *[free text]*
repeat subfields for each of the target lesions selected	Size	• Largest dimension on axial plane (mm) *[Numeric]*<br>• Dimension of the axis perpendicular to the largest diameter (mm) *[Numeric]*
	Reference image	Number of reference image *[Numeric]*<br>(with automatic link to key image)
	CT appearance	• Structure<br>○ areas of contrast enhancement *[free text]*<br>○ areas of necrosis/colliquation *[free text]*<br>○ other *[free text]*<br>• Relationship with neighboring structures *[free text]*

**Bulky disease**		• Complications *[free text]*
	Notes	*[free text]*
	Site	• Chest<br>• Abdomen<br>• Other *[free text]*
	Largest dimension	AP (mm) x LL (mm) x CC (mm) diameters *[Numeric]*
	Reference image	• Number of reference image *[Numeric]* (with automatic link to key image)<br>• AP (mm) x LL (mm) x CC (mm) diameters on reference image *[Numeric]*
	CT appearance	• Structure<br>o areas of contrast enhancement *[free text]*<br>o areas of necrosis/colliquation *[free text]*<br>• Relationship with neighboring structures<br>o airways (displacement / compression / infiltration)<br>o vessels (displacement / compression / infiltration)<br>o other *[free text]*<br>• Complications<br>o airway compression<br>o vascular compression<br>o vessel thrombosis<br>o other *[free text]*
	Notes	*[free text]*
**Spleen**	Largest dimension<br>(measured on longitudinal MPR)	• normal (<12cm)<br>• borderline high (12-13cm)<br>• splenomegaly (>13cm)
	Structure	• homogeneous<br>• inhomogeneous/micronodular<br>• focal lesions *[Numeric]***
	*if focal lesions, specify for up to 2 target lesions:	• Largest dimension on axial plane (mm) *[Numeric]*<br>• Dimension of the axis perpendicular to the largest diameter (mm) *[Numeric]*<br>• Number of reference image *[Numeric]* (with automatic link to key image)
**Extranodal disease**<br>***open subfields for each extranodal site selected)***	**Site**<br>**(up to 2 target lesions can be selected)**	• Limited disease (stage I-II) / advanced disease (stage III-IV)<br>o liver<br>o kidney<br>o lung

		○ GI tract<br>○ muscles/skin<br>○ orbit<br>○ CNS<br>○ bone<br>○ other extranodal sites
	Liver	• Hepatomegaly (Yes/No)<br>○ If Yes: homogeneous / inhomogeneous<br>• Focal lesions *[Numeric]**<br>• CT appearance *[free text]*
	*if focal lesions, specify for up to 2 target lesions:	• Largest dimension on axial plane (mm) *[Numeric]*<br>• Dimension of the axis perpendicular to the largest diameter (mm) *[Numeric]*<br>• Number of reference image *[Numeric]* (with automatic link to key image)
	Kidney	• Unilateral / bilateral involvement<br>• Hilar / perirenal / extrarenal involvement<br>• Focal lesions *[Numeric]**<br>• CT appearance *[free text]*
	*if focal lesions, specify for up to 2 target lesions:	• Largest dimension on axial plane (mm) *[Numeric]*<br>• Dimension of the axis perpendicular to the largest diameter (mm) *[Numeric]*<br>• Number of reference image *[Numeric]* (with automatic link to key image)
	Lung	• Site of lung involvement (right/left, lobe, segment)<br>• CT appearance<br>○ Single mass (if Yes: Largest dimension on axial plane (mm) *[Numeric],* Dimension of the axis perpendicular to the largest diameter (mm) *[Numeric]*, Number of reference image *[Numeric]* (with automatic link to key image)<br>○ Multiple lesions (if Yes: Largest dimension on axial plane (mm) *[Numeric],* Dimension of the axis perpendicular to the largest diameter (mm) *[Numeric]* for up to 2 target lesions, Number of reference image *[Numeric]* (with automatic link to key image)<br>○ Consolidation<br>○ Interstitial involvement<br>○ Parenchymal infiltration by lymphoadenopathies<br>○ structure *[free text]*<br>○ other *[free text]*

	GI tract	• Site of GI involvement (stomach, duodenum, jejunum, ileum, cecum/appendix, colon/rectum)<br>• Focal lesions (if Yes: Largest dimension on axial plane (mm) *[Numeric],* Dimension of the axis perpendicular to the largest diameter (mm) *[Numeric]* for up to 2 target lesions, Number of reference image *[Numeric]* (with automatic link to key image)<br>• CT appearance<br>○ vegetating lesion<br>○ ulcerated lesion<br>○ stenosing lesion<br>○ structure *[free text]*<br>○ other *[free text]*
	Muscles/skin	• Site of lesions *[free text]*<br>• Focal lesions (if Yes: Largest dimension on axial plane (mm) *[Numeric],* Dimension of the axis perpendicular to the largest diameter (mm) *[Numeric]* for up to 2 target lesions, Number of reference image *[Numeric]* (with automatic link to key image)<br>• CT appearance *[free text]*
	Orbit	• Site of lesions *[free text]*<br>• Focal lesions (if Yes: Largest dimension on axial plane (mm) *[Numeric],* Dimension of the axis perpendicular to the largest diameter (mm) *[Numeric]* for up to 2 target lesions, Number of reference image *[Numeric]* (with automatic link to key image)<br>• CT appearance *[free text]*
	CNS	• Site of lesions<br>○ brain lobes<br>○ brainstem<br>○ cerebellum<br>○ other *[free text]*<br>• Focal lesions (if Yes: Largest dimension on axial plane (mm) *[Numeric],* Dimension of the axis perpendicular to the largest diameter (mm) *[Numeric]* for up to 2 target lesions, Number of reference image *[Numeric]* (with automatic link to key image)<br>• CT appearance *[free text]*
	Bone	• Site of lesions *[free text]*<br>• Focal lesions (if Yes: Largest dimension on axial plane (mm) *[Numeric],* Dimension of the axis perpendicular to the largest diameter (mm)

		[Numeric] for up to 2 target lesions within soft tissue components, Number of reference image *[Numeric]* (with automatic link to key image)<br>● CT appearance *[free text]*
	Other extranodal sites	● Site of lesions *[free text]*<br>● Focal lesions (if Yes: Largest dimension on axial plane (mm) *[Numeric],* Dimension of the axis perpendicular to the largest diameter (mm) *[Numeric]* for up to 2 target lesions, Number of reference image *[Numeric]* (with automatic link to key image)<br>● CT appearance *[free text]*
**Non-measurable lesions**	Yes/No<br>If Yes:	● Pleura<br>○ effusion (thickness) *[Numeric]*<br>○ nodules/thickening *[free text]*<br>● Pericardium<br>○ effusion (thickness) *[Numeric]*<br>○ nodules/thickening *[free text]*<br>● Peritoneum<br>○ effusion (thickness) *[Numeric]*<br>○ nodules/thickening *[free text]*<br>● CT appearance *[free text]*
**SELECTED TARGET LESIONS (TL)**<br>**(note: n≥6 in diffuse disease)**<br>***Repeat fields for n times***	TL(n)*	● Nodal (N) / extranodal disease (E)*<br>● Site*<br>● Number of reference image *[Numeric]* (with automatic link to key image)<br>● Size and CT appearance*
**TOTAL LESION BURDEN**	Sum Product Diameter	*(mm) [Numeric]*
	Volume of target lesions	*(mm^3) [Numeric]*
**COMPLICATIONS**		● Thrombosis<br>● Pneumonia<br>● Other *[free text]*
**INCIDENTAL FINDINGS**		*[free text]*
**NOTES**		*[free text]*

**CONCLUSIONS**<br>**(note: according to Lugano revision of Ann Arbor criteria)**		● Limited disease / Advanced disease<br>● Stage I-II / III-IV<br>● Other *[free text]*

IMAGES

FIELD	DETAIL	ADMITTED VALUES
**Key images**	Key images	*[Image]*
